# Adherence of *Lactobacillus salivarius* to HeLa Cells Promotes Changes in the Expression of the Genes Involved in Biosynthesis of Their Ligands

**DOI:** 10.3389/fimmu.2019.03019

**Published:** 2020-01-09

**Authors:** Carla Martín, Iván Fernández-Vega, Juan E. Suárez, Luis M. Quirós

**Affiliations:** ^1^Área de Microbiología, Universidad de Oviedo, Oviedo, Spain; ^2^Instituto Universitario Fernández-Vega, Universidad de Oviedo, Oviedo, Spain; ^3^Instituto de Investigación Sanitaria del Principado de Asturias, Oviedo, Spain; ^4^Hospital Universitario Central de Asturias, Oviedo, Spain

**Keywords:** bacterial adherence, glycosaminoglycan, OppA, *Lactobacillus*, proteoglycan, heparan sulfate, chondroitin sulfate

## Abstract

The attachment of a variety of *Lactobacilli* to the mucosal surfaces is accomplished through the interaction of OppA, a superficial bacterial protein also involved in oligopeptide internalization, and the glycosaminoglycan moiety of the proteoglycans that form the epithelial cell glycocalyx. Upon the interaction of the vaginal isolate *Lactobacillus salivarius* Lv72 and HeLa cell cultures, the expression of *oppA* increased more than 50-fold over the following 30 min, with the overexpression enduring, albeit at a lower rate, for up to 24 h. Conversely, transcriptional analysis of 62 genes involved in proteoglycan biosynthesis revealed generalized repression of genes whose products catalyze different steps of the whole pathway. This led to decreases in the superficial concentration of heparan (60%) and chondroitin sulfate (40%), although the molecular masses of these glycosaminoglycans were higher than those of the control cultures. Despite this lowering in the concentration of the receptor, attachment of the *Lactobacilli* proceeded, and completely overlaid the underlying HeLa cell culture.

## Introduction

The organisms included in the genus *Lactobacillus* belong to the Filum Firmicutes. They are anaerobic, usually aerotolerant, bacteria that ferment sugars to lactic and other organic acids which are also auxotrophic for many essential nutrients. The genus comprises 241 species, according to the List of Prokaryotic Names with Standing in Nomenclature (July, 2019) and it is polyphyletic, to the extent that its division into 10 or 16 different genera, on the basis of their genome characteristics, has been proposed (1–3). *Lactobacilli* occupy many different habitats, ranging from the physical environment, where they behave as saprophytes, to the fact that they constitute a substantial part of the starter microbiota involved in food and feed fermentation, as well as being present within human body cavities, where they are an important part of the autochthonous microbiota. In the latter scenario, the lactic acid, H_2_O_2_ and bacteriocins produced by resident *Lactobacilli* protect the internal cavities from infection, while enhancing immune system maturation and tightening the boundaries between the epithelial cells that line the mucosa. This “microbial antagonism” is also based on *Lactobacilli*'s specific adherence to the mucosal surfaces, where they form biofilms that preclude pathogens coming into contact with epithelial cells (4–6).

There is some degree of specificity between the different body cavities and the species of *Lactobacilli* that thrive in each of them, and this preference depends not only on environmental conditions, but also on the ability of the bacterium to adhere to each mucosal surface (7). Attachment depends on the specific recognition between adhesins located on the exterior of the bacteria and the receptors that protrude from epithelial cells, and a variety of adhesins have been described for *Lactobacilli* (8–11). In addition, a variety of surface proteins have been found to act as adhesins, such as those that bind to mucus through the so called Mub-repeats (12), some of which depend on sortase-driven anchoring to the bacterial surface (13). Finally, some cytoplasmic proteins appear to reach the bacterial surface and behave as adhesins, in spite of them not presenting discernible membrane-translocating motives. Among them are the glycolytic enzymes glyceraldehyde 3-P-dehydrogenase (14, 15), enolase (16), and pyruvate dehydrogenase (17) and the protein synthesis factors EF-Tu (18) and GroEL (19).

The receptors to which *Lactobacilli* adhesins attach are part of the cells or the extracellular matrix present in the epithelium. The latter is made of polysaccharides (hyaluronic acid), fibrillar proteins of the collagen family and fibronectin (5, 20), and glycoproteins, with mucins and proteoglycans (PGs) being the most abundant. PGs are complex macromolecules whose core is made of specific proteins that, in turn, determine their location—either in the cell or at the extracellular matrix—and is covalently bound to glycosaminoglycans (GAGs). These are linear heteropolysaccharides consisting of repeating disaccharide units made of amino and uronic monosaccharides or galactose to which sulfate groups may be attached (21). Heparan sulfate proteoglycans (HSPGs) are usually the most prevalent GAG at the cell surface and in the pericellular matrix, and their structures may include not only heparan sulfate (HS), but also chondroitin sulfate (CS) moieties. Synthesis of HS and CS chains occurs mainly in the Golgi apparatus, and starts by the joining of a xylose to a specific serine residue on the core protein, followed by the successive addition of two galactoses and one glucuronic acid. The addition of the following residue determines the type of GAG that will be synthesized: *N*-acetylglucosamine will produce HS, while *N*-acetylgalactosamine leads to CS. The elongation of the chain is catalyzed by a series of enzymes that specifically recognize the sugars to be incorporated and act in a coordinated fashion. Finally, discrete regions of the polysaccharide may be modified through various reactions, including *N*-sulfation, epimerization and various *O*-sulfations (21). The specific combination of reactions that take place on each disaccharide gives rise to molecules with great structural diversity, resulting in them being able to interact with many biological ligands by means of the high affinity sequences they have for them. These interactions make PGs essential in the control of many biological processes, including organogenesis, cell junction, cell signaling or wound healing, among other functions (22).

In previous communications we reported that soluble GAGs antagonized the attachment of *L. salivarius* Lv72 and other *Lactobacilli* to epithelial cell cultures. Moreover, we found that heparin recognized a component of the external proteomes of *Lactobacilli* that turned out to be the oligopeptide-binding protein OppA (23), which is the surface component of an ATP-binding cassette (ABC) previously described as being involved in oligopeptide internalization (24). OppA modeling revealed the presence of a groove on its surface whose diameter matched the width of GAG-chains. The introduction of mutations on triplets encoding positively charged amino acids located on the vicinity of the groove blocked binding, thus confirming the role of OppA as a *Lactobacilli* adhesin, and that of GAGs, especially HS, as being its receptor on the mucosal surface (23–26).

These data suggest that the mutualistic relation established between mucosal surfaces and resident *Lactobacilli* is partially dependent on the specific interaction between OppA and the GAGs that cover the epithelial cells, mainly HS chains. Given this premise, we postulated that contact between the two cell types might induce changes in the expression of the genes encoding the bacterial adhesins, thus affecting their superficial concentrations. Moreover, considering that cells exercise exquisite control over both the composition and sequencing of HSPG in response to physiological and pathological changes, these changes might occur as part of the response of the epithelial cells to their interaction with the microbiota. This might result in tightening the bacterial and epithelial layers and in the efficient exclusion of undesirable microorganisms. The data obtained from the analysis of the molecular events resulting from the contact of both cell types are reported in this communication.

## Materials and Methods

### Bacterial Strain, Eukaryotic Cell Line, and Growth Conditions

*Lactobacillus salivarius* Lv72 and HeLa cell cultures (ATCC CCL-2) were propagated in MRS medium (Becton, Franklin Lakes, USA) and Dulbecco's Modified Eagle's minimal essential medium (DMEM) (GibcoBRL, Eragny, France) supplemented with 10% (w/v) fetal bovine serum (GibcoBRL), respectively, as previously described (23).

### Total RNA Isolation From Pure and Mixed *L. salivarius* Lv72/HeLa Cell Cultures and cDNA Synthesis

Confluent HeLa cell cultures in 25 cm^2^ tissue culture flasks (Nunc, Roskilde, Denmark) were washed twice with DMEM and a suspension of freshly prepared exponentially growing *L. salivarius* Lv72 in the same medium was added (10^7^ cells/ml, final concentration) and incubated for 1 h at 37°C under a 5% CO_2_ atmosphere. Controls were treated in the same way except that only the sterile medium was added in the final step. The supernatants were discarded, and the cell cultures were washed twice with DMEM and overflowed with 12 ml of the same medium. Samples were taken at 10, 20, and 30 min and at 1, 2, 4, 6, and 24 h and subjected to RNA extraction using the RNeasy kit (Qiagen; Hilden, Germany), following the manufacturer's specifications. To ensure removal of residual contaminating DNA, the samples were subjected to treatment with RNase-free DNase. The concentration of RNA was determined by measuring the absorbance at 260 nm. Aliquots of the samples were stored at −80°C until their future use. Synthesis of cDNA was carried out using the High Capacity cDNA Transcription Kit (Applied BioSystems; Foster City, CA) following the manufacturer's instructions. The reactions were performed in an iCycler IQ thermocycler (BioRad; Hercules, CA) using 2 μg RNA as substrate. The reaction products were cleaned using the PCR Clean-Up GenElute kit (Sigma-Aldrich, St. Louis, USA) as recommended by the provider. Finally, the aliquots containing the cDNA were diluted 1:20 with water and stored at −20°C until use. The data on eukaryotic gene expression throughout this paper were obtained from 24 h post-exposition samples since no significant differences compared to controls could be detected after shorter periods.

### qRT-PCR Reactions

qRT-PCR reactions, and analysis of amplimer products were carried out according to the methods already detailed (27). Primers corresponding to the human and *Lactobacilli* versions of the glyceraldehyde 3-P-dehydrogenase genes were included on each plate as controls to monitor run variations and to normalize individual gene expression. The primer sequences used are detailed in Supplemental Table 1. The comparison of the individual sets of results corresponding to each experiment with respect to the results of its corresponding control was carried out using a Mann-Whitney *U*-test.

### Immunohistochemistry

HeLa cells were propagated on culture microscope slides under the conditions described above. After incubation for 24 h, the cultures were washed three times with phosphate buffered saline (PBS), fixed with acetone for 20 min at −20°C, washed with the same buffer and incubated overnight at 4°C with appropriate dilutions of the primary antibodies (Table 1). The slides were then washed for 30 min with PBS, placed in the dark and incubated with the secondary antibodies (Table 1) for 90 min in a humid chamber. The samples were washed three times with PBS and incubated successively with 1 μg/ml phalloidin-TRITC conjugate (Sigma-Aldrich) for 90 min and 10 ng/ml DAPI (Southern Biotech; Birmingham, USA). The preparations were visually examined and photographed in a Leica DMR-XA fluorescence microscope coupled to Leica Qfluoro software in the Image Processing facility of the University of Oviedo. The quantification of fluorescence for the subsequent statistical analysis was carried out using ImageJ analysis software (28).

**Table 1:** Antibodies and dilution used.

**Antigen**	**Species of origin**	**Dilution**	**Supplier**
Syndecan 1 (CD138)	Mouse	1:100	Dakocytomation
Syndecan 2	Rabbit	1:250	Santa cruz biotechnology
Syndecan 3	Goat	1:50	Santa cruz biotechnology
Glypican 1	Rabbit	1:100	Thermoscientific
Perlecan	Rabbit	1:100	Santa cruz biotechnology
Agrin	Goat	1:100	Santa cruz biotechnology
TGFβ RIII	Mouse	1:100	Santa cruz biotechnology
HS (10E4 epitope)	Mouse	1:100	Amsbio
CS (Clone CS-56)	Mouse	1:100	Sigma-aldrich corp
OppA rabbit	Rabbit	1:100	Obtained from our own lab
Alexa Fluor 488	Goat anti-rabbit	1:200	Invitrogen
Alexa Fluor 488	Donkey anti-mouse	1:500	Invitrogen
Cy3	Donkey anti-mouse	1:50	Jackson immunoresearch laboratories
Cy3	Monkey anti-goat	1:100	Santa cruz biotechnology

### Adherence Assays

HeLa cell cultures grown on microscope slides were washed three times with DMEM on its own, after which a suspension of exponentially growing *L. salivarius* Lv72 suspended in the same medium was added to the slides (10^9^ bacteria/ml, final concentration) and they were incubated for up to 24 h at 37°C under a 5% CO_2_ atmosphere in a humid chamber. The supernatant was discarded, the slides were washed twice with PBS and the degree of adherence was established using immunochemical detection (see above) using OppA-specific primary antibodies.

### Purification and Determination of GAGs

For the extraction of GAGs, HeLa cell cultures were kept pure or in contact with *L. salivarius* Lv72 for 24 h as explained above. After removing the medium by aspiration, the cell monolayers were washed with PBS. Next, 6 ml of 50 mM Tris-HCl buffer pH 8 containing 6 M guanidine chloride (Sigma-Aldrich) and 3 mM dithiothreitrol (DTT) (Sigma-Aldrich) were added and incubated with stirring at 60°C for 1 h. Subsequently, 15 ml of 50 mM Tris-HCl pH 8 containing 6.7 mM calcium chloride (Merck) and 50 μl of 1 mg/ml proteinase K (Sigma-Aldrich) were added, and the contents of the plates were extracted and incubated at 56°C for 16 h. GAGs were precipitated with 85% ethanol for a minimum of 2 h at −80°C, and collected by centrifugation at 4,000 rpm for 30 min at 4°C. The sediments were dried and resuspended in 2 ml of 10 mM phosphate buffer pH 6.8 containing 5 mM CaCl_2_ and 20 μl of 1 mg/ml DNAse (Sigma-Aldrich), followed by incubation for 4 h at 37°C. Then, NaOH and NaBH_4_ were added to the extracts to a final concentration of 0.2 M and 50 mM, respectively, and they were incubated at room temperature for 18 h. Next, the pH was equilibrated with 500 μl of 2 M HCl and 200 μl of 1 M sodium acetate for each ml of solution, and the samples were centrifuged at 4,000 rpm for 30 min at 4°C. The supernatant was collected, and the GAGs were precipitated again with 85% ethanol and resuspended in H_2_O.

The purification of HS and CS chains was carried out by digestion with bacterial lyases. The CS was obtained by digesting the mixture of GAGs overnight at 37°C with a mixture of heparinase I, II, and III (Sigma-Aldrich) at a final concentration of 500 mU/ml each, in 0.1 M sodium acetate buffer pH 6.8 containing 10 mM NaCl. The HS was isolated by degradation with chondroitinase ABC (Sigma-Aldrich) at a final concentration of 250 mU/ml in 50 mM Tris-HCl buffer pH 8 for 3 h at 37°C. In both cases, the resulting polysaccharide chains were obtained by precipitation with 85% ethanol at −80°C for 2 h.

The determination of GAG concentrations was carried out through spectrophotometry of their adducts with 1,9-dimethyl-methylene blue as previously reported (29).

### GAG Analysis by Molecular Exclusion Chromatography

GAGs were labeled with 0.1 mg/ml FITC in 0.1 M sodium carbonate buffer pH 9, for 18 h at 4°C in the dark with shaking (30). Unreacted FITC was removed by precipitation with 85% ethanol for 2 h at −80°C, followed by centrifugation at 4,000 rpm for 20 min at 4°C. The sediment was resuspended in 0.1 M sodium carbonate buffer pH 9, and the precipitation was repeated until no FITC residues remained in the supernatant. Finally, the precipitate was resuspended in 300 μl of 50 mM phosphate buffer pH 7.2 containing 150 mM NaCl. Two-hundred microliter of each sample was subjected to molecular exclusion chromatography using a 10/300 Superose 12 column previously equilibrated in 50 mM phosphate buffer pH 7.2 and 150 mM NaCl, connected to a FPLC ÄKTA Design system (GE Healthcare, Chicago, USA). The column was eluted with a flow of 0.3 ml / min, and 0.5 ml fractions were collected. Aliquots of 350 μl of each of the fractions were added to a fluorescence plate (Nunc). Fluorescence was measured in a PerkinElmer LS55 fluorimeter (PerkinElmer, Waltham, Massachusetts, U.S.A), using wavelengths of 488 nm for excitation and 560 nm for emission.

## Results

### Differential Expression of the Genes That Encode the Proteoglycan Core Proteins

Our previous studies have shown that HS chains present in HeLa cells play a prominent role in its interaction with *Lactobacilli* adhesins and the consequent adherence of the microorganism. Only a limited number of genes encode the core proteins of HSPGs, three of which, perlecan, agrin and collagen, encode molecules located in the extracellular matrix. The remaining HSPGs are all molecules located in the cell, mostly on the cell surface, although serglycin is found intracellularly.

**Figure 1:**
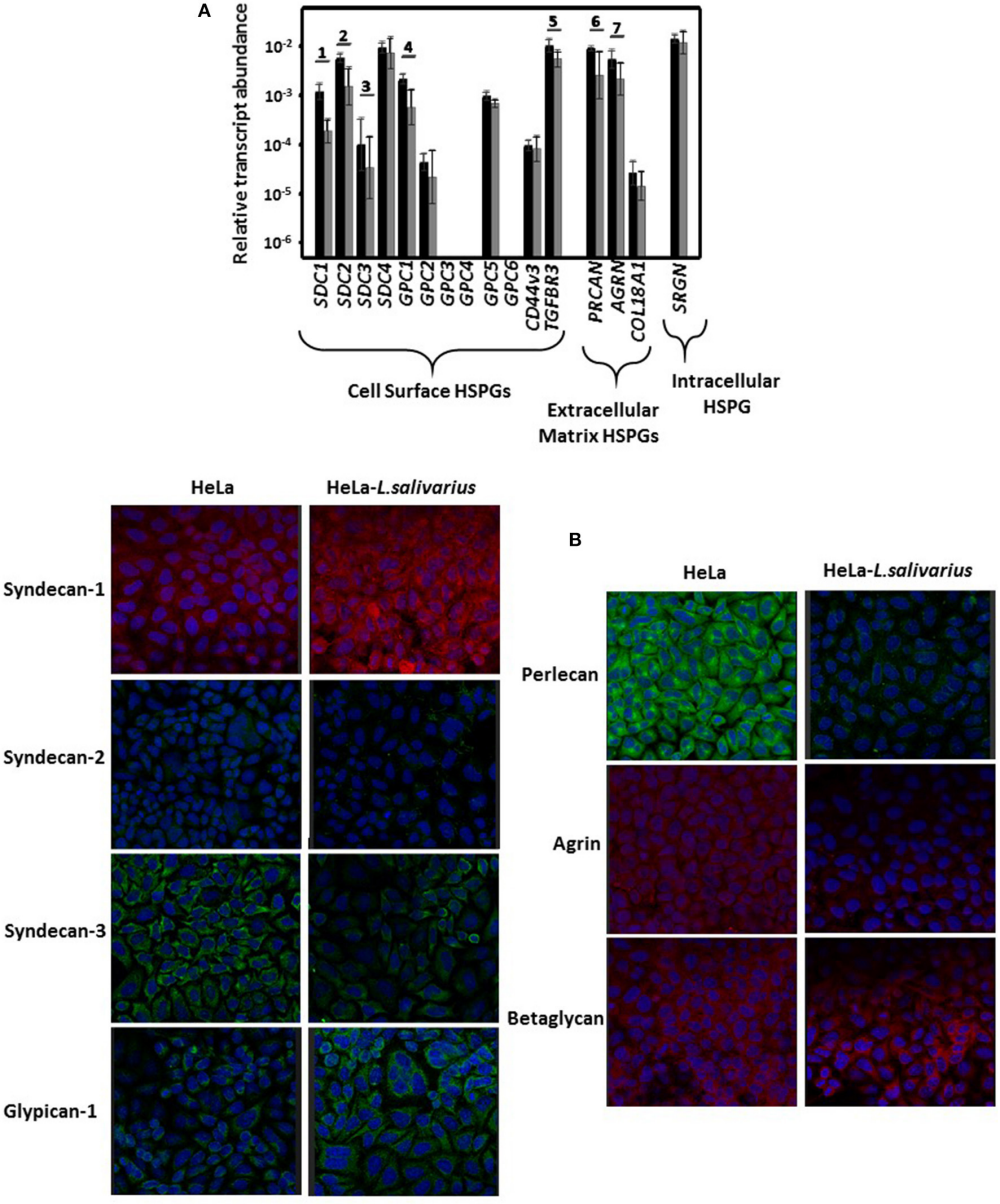
Differential expression of the proteoglycan core proteins. **(A)** Differential expression of the genes that encode the PG core proteins of HeLa cells in pure culture (black bars) and after having been incubated for 1 h with *L. salivarius* Lv72 (gray bars). Note that the ordinates scale is logarithmic. Statistically significant differences (*p* < 0.01) are indicated by numbers. The data are the combined results of at least four independent determinations. **(B)** Immunolocalization of PGs in pure HeLa cell cultures (left) or those previously incubated with *L. salivarius* (right). The quantification of fluorescence using ImageJ analysis software and subsequent statistical analysis gave rise to significant results for syndecan 1 (*p* < 0.01), syndecan 2, syndecan 3, glypican 1, perlecan and agrin (*p* < 0.001 in all cases), but not for betaglycan (*p* = 0.1).

Analysis of the core protein transcrips synthesized by HeLa cells in either pure culture or after their interaction with *L. salivarius* Lv72 (mixed cultures), revealed no expression of genes *GPC3, GPC4*, and *GPC6* among those that encode glypican isoforms. Conversely, the genes *GPC1, GPC2*, and *GPC5* were expressed under both conditions, although *GPC1* mRNA appeared underexpressed around 70% in mixed with respect to pure HeLa cell cultures. All four genes encoding syndecans were found to be expressed, although with significant reductions of 80, 70, and 70% for *SDC1, SDC2*, and *SDC3*, respectively in mixed cultures. Similar expression attenuations were found for the genes that encode the core proteins of perlecan (*PRCAN*), agrin (*AGRN*), and betaglycan (*TGFBR3*), while no changes were evidenced in the expression of *COL18A1* (collagen XVIII), *CD44v3* (CD44 isoform 3), and *SRGN* (serglycin) (Figure 1A).

When some of these changes were analyzed by immunohistochemistry, it was observed that the label intensity of syndecan 2, syndecan 3, glypican 1, perlecan and agrin decreased, the results being statistically significant (*p* < 0.001 in all cases). This therefore confirmed that differences in transcription correlated with net decreases in protein levels. In the case of betaglycan, no significant staining difference was observed in the presence of the microorganism (*p* = 0.1). However, in contrast to what was observed at the transcription level, the immunostaining of syndecan 1 significantly increased after the adhesion of lactobacillus (*p* < 0.01) (Figure 1B).

### Comparison Between the Expression of the Determinants That Encode GAG Polymerization Enzymes

**Figure 2:**
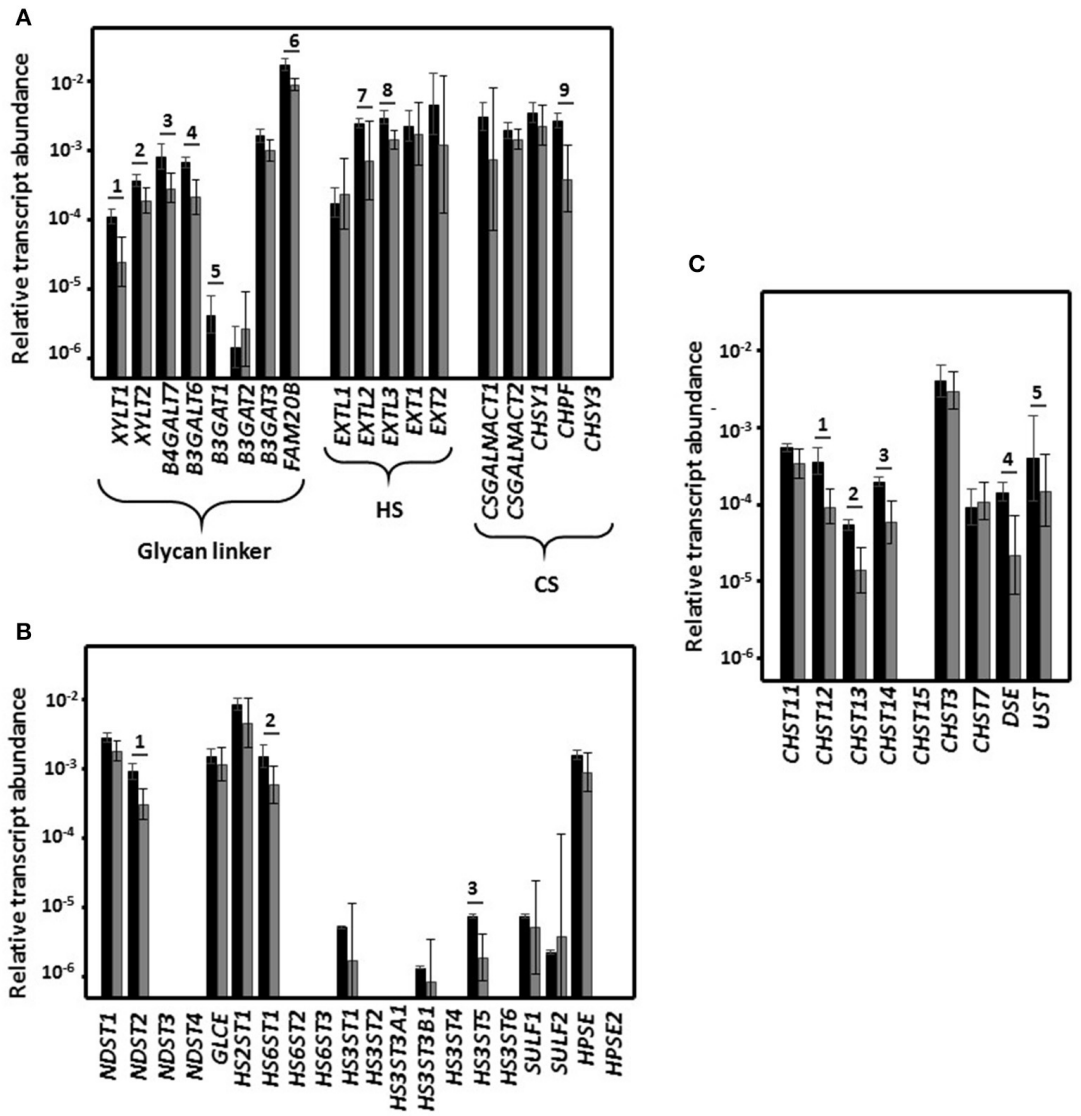
Differential expression of the genes that encode the enzymes involved in the biosynthesis of GAGs. **(A)** Differential expression of genes encoding glycosyltransferases involved in common linkage region sequence and GAG chain synthesis. **(B)** Differential expression of genes involved in the modification of HS chains. **(C)** Differential expression of genes involved in the modification of CS chains. Relative transcript abundance of mRNAs for HeLa pure culture (black bars) and after HeLa cells were incubated for 1 h with *L. salivarius* Lv72 (gray bars) are plotted on a log scale for each gene assayed and the spreads represent standard deviations. Statistically significant differences (*p* < 0.01) are indicated by numbers. The data are the result of at least four independent determinations.

The proteins encoded by *XYLT1* and *XYLT2* catalyze the union of a xylose residue to the hydroxyl group of specific serine residues that form part of the core protein. This xylose unit can be phosphorylated by the product of *FAM20*, which appears to be involved in regulation of GAG synthesis. Next, biosynthesis continues through the successive addition of two galactose residues, both reactions being catalyzed by enzymes encoded by *B4GALT7* and *B4GALT6*. Finally, a tetrasaccharide, typical of HS and CS, is formed through the linking of glucuronic acid, which is mediated by the products of any of the three isoforms of *B3GAT1-3*, although in this study it was mainly transcripts of *B3GAT3* that were detected. Transcription of all these genes, with the exception of *B3GAT3*, were reduced by between 50 and 90% in HeLa cell cultures previously incubated with *L. salivarius* Lv72 (Figure 2A).

Further polymerization, in the case of HS, depends on the activity of an *N*-acetylglucosamine transferase (EXTL1-3) and of the copolymerases 1 and 2 (EXT1 and EXT2), which incorporate alternating glucuronic acid and *N-*acetylglucosamine residues to the growing chain. In the case of CS, elongation is initiated by the incorporation of an *N*-acetylgalactosamine (CSGALNACT1-2) followed by alternate additions of glucuronic acid and *N*-acetylgalactosamine, which are catalyzed by CS synthetase 1 and 3 (CHSY1 and CHSY3) and enhanced by the CS polymerization factor (CHPF). While expression of most of these genes was not significantly changed as a function of the contact of *L. salivarius* Lv72 with the HeLa cell cultures, the transcript concentrations of *EXTL3* and *EXTL2* and *CHPF* from the polymerization routes of HS and CS dropped by 50 and 70%, respectively (Figure 2A).

### Differential Expression of the Genes That Mediate HS Modification

The fine structure of HS can change through *N*-deacetylation/*N*-sulfation in reactions catalyzed by bifunctional *N*-deacetylases/*N*-sulfotransferases encoded by genes *NDST1* to *NDST4*. In addition, glucuronic acid epimerization may generate iduronic acid (GLCE), which is sometimes followed by *O*-sulfation in position 2 of this residue (HS2ST1), and *O*-sulfations in positions 6 (HS6ST1 to HS6ST3) and 3 (HS3ST1 to HS3ST6) of the glucosamine residue. Following export from the cell, HS can be processed by heparanase (HPSE), an endo-β-D-glucuronidase that generates 10–20 residue oligosaccharides; a second isoform exists (HPSE2) which has no enzymatic activity but does have regulatory capacity. HS chains can also be desulfated through the action of two extracellular sulfatases (SULF1, SULF2).

Of the 20 genes involved in HS structural fine-tuning, almost half were not expressed by the HeLa cell cultures under the experimental conditions in this work. Most of the remainder did not significantly change their expression level in response to contact with *L. salivarius* Lv72, although a reduction was observed for three genes, namely *NDST2, HS6ST1*, and *HS3ST5* (Figure 2B).

### Differential Expression of the Genes That Mediate Chondroitin Sulfate Modification

The reactions that lead to CS diversification include 4-*O*-sulfation (CHST11 to CHST14) and 6-*O*-sulfation (CHST3, CHST7, and CHST15) of *N*-acetylgalactosamine, epimerization of the glucuronic acid in position 5 to iduronic acid (DSE) to give dermatan sulfate, and 2-*O*-sulfation of this residue (UST). The expression of five of these nine determinants was lower when HeLa cells had been in contact with the *Lactobacilli*, the drop ranging from 60 to 75% (Figure 2C).

### Characterization of Glycosaminoglycans as a Function of the Interaction Between HeLa Cells and *L. salivarius* Lv72

**Figure 3:**
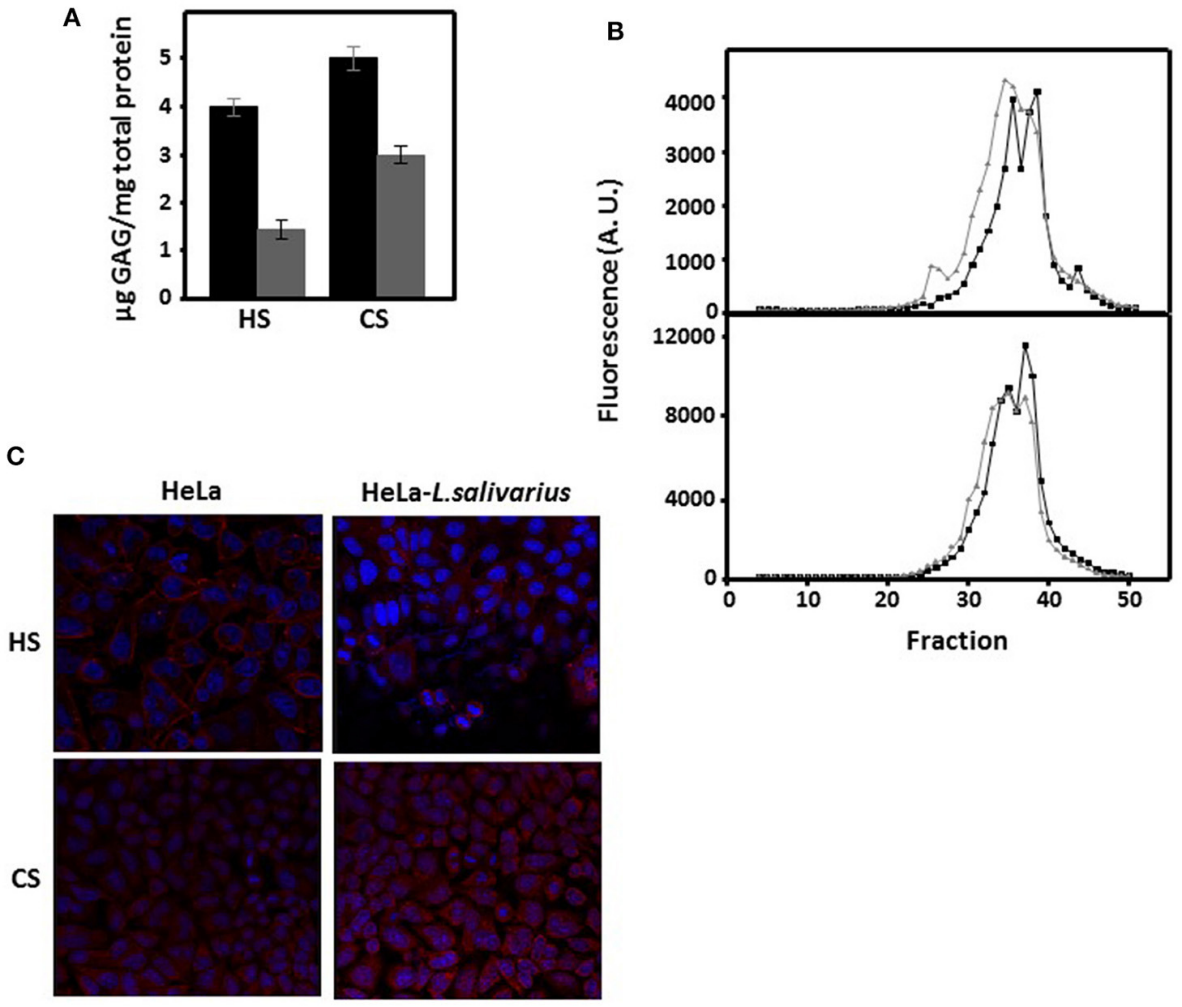
Characterization of GAGs as a function of the interaction between HeLa cells and *L. salivarius* Lv72. **(A)** Quantification of HS and CS extracted from the surface of pure HeLa cell cultures (black bars) or those previously incubated with *L. salivarius* Lv72 (gray). The differences are statistically significant (*p* < 0.001 for HS) and (*p* < 0.01 for CS). The data are the result of at least four independent determinations. **(B)** Molecular exclusion chromatography of the HS (upper panel) and CS (bottom panel) chains extracted from the surface of pure HeLa cell cultures (black lines) or those previously incubated with *L. salivarius* Lv72 (gray). **(C)** Immunolocalization of HS and CS chains in pure HeLa cell cultures (left) or those previously incubated with *L. salivarius* Lv72 (right).

The alterations observed in the expression of the genes responsible for the synthesis of GAGs in HeLa cells that had been in contact with *L. salivarius* Lv72 suggest that both the quantitative levels of these saccharide chains and their structural features (chain size and sulfation pattern) might be affected. To carry out quantifications, GAGs were extracted from cell cultures and their concentrations were determined through the spectrophotometry of their adducts using 1,9-dimethyl-methylene blue. The results showed significant reductions of more than 60% for HS and close to 40% for CS after the HeLa cell cultures were incubated with the bacterium (Figure 3A). Chain size characterization was performed by molecular exclusion chromatography. The data obtained showed a shift toward higher molecular weights, with the change for HS being greater than that for CS (Figure 3B).

The visualization of the chains of both GAGs in cell cultures was carried out by immunohistochemistry, using monoclonal antibodies against specific epitopes. 10E4 is a native HS epitope that includes *N*-sulfated glucosamine residues, and the monoclonal antibody CS-56, which was used to detect CS chains, reacts preferentially with CS-D (sulfated at C-2 and C-6) although it is also able to recognize other types of structures, including CS-A, -C, and -E (31). The results showed a decrease in the immunolabelling of HS after contact with the *Lactobacilli* (*p* < 0.05), while in the case of CS, no significant differences were observed (*p* = 0.12) (Figure 3C).

### Differential Expression of *L. salivarius* Lv72 *oppA*

**Figure 4:**
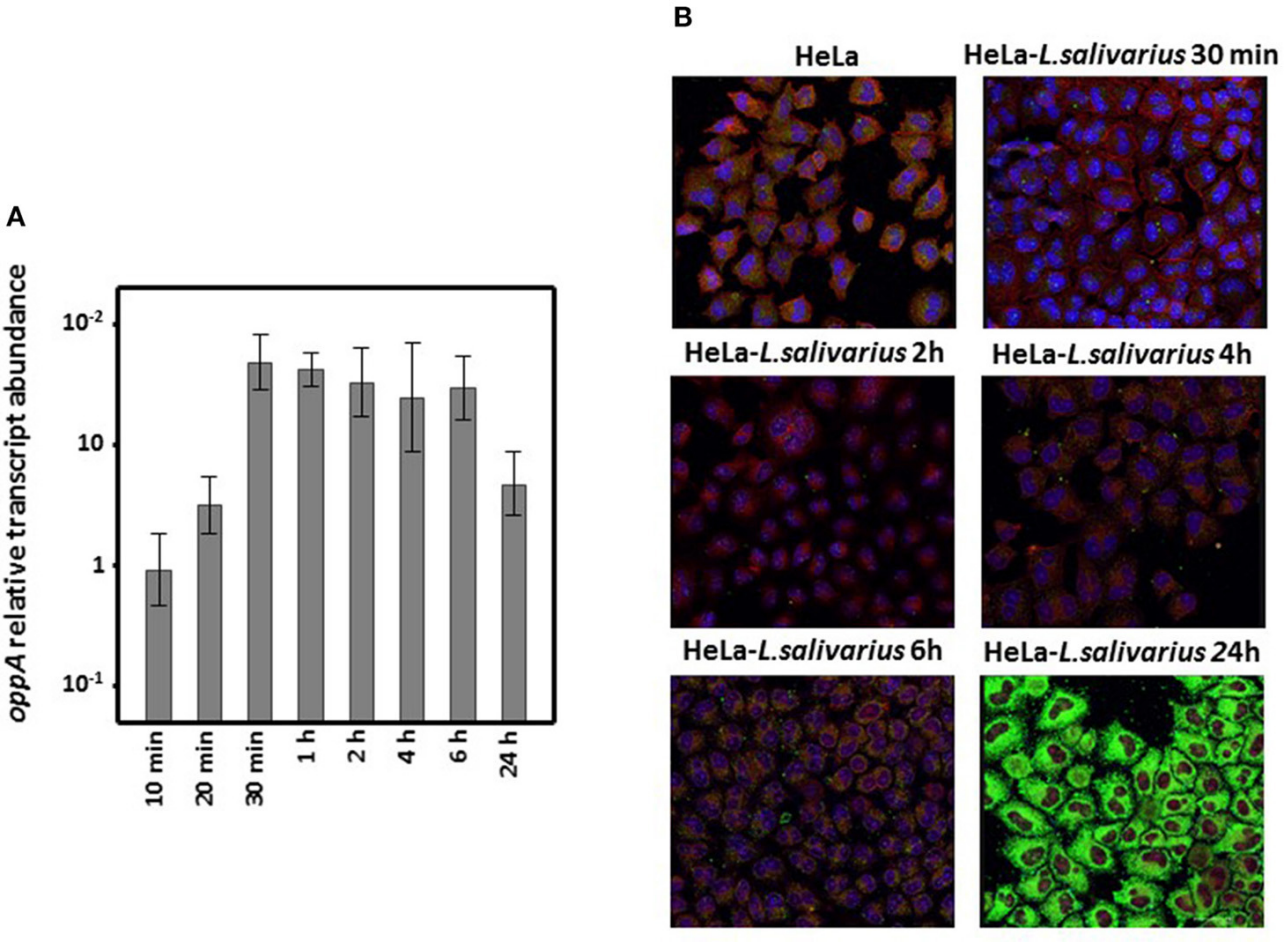
Differential expression of *L. salivarius* Lv72 *oppA*. **(A)** Increase in *oppA*-specific RNA accumulation when extracted from mixed HeLa cell-*L. salivarius* Lv72 vs. pure bacterial cultures. The ordinates scale is logarithmic and standard deviations are indicated by spreads. The data are based on at least four independent determinations. **(B)** Immunolocalization of OppA in mixed HeLa cell *L. salivarius* Lv72 cultures. OppA antibody binding, nuclei and actin were revealed with Alexa 488 (green), DAPI (blue), and phalloidin (red), respectively.

Interaction with HeLa cell cultures provoked the sustained enhancement of *oppA* expression by *L. salivarius* Lv72 with values reaching a more than 50-fold increment after between 30 min and 6 h of co-incubation. Even 24 h later the transcription of *oppA* from the *Lactobacilli* was several times higher than in pure bacterial cultures (Figure 4A).

*L. salivarius* does not proliferate in DMEM devoid of bovine fetal serum, as evidenced by the lack of increase over time in the viable counts of the cultures or the phenol red pH-dependent color change. The presence of OppA on its surface and the subsequent adherence of *L. salivarius* to HeLa cell cultures was followed by immunochemical detection for 24 h using OppA-specific as primary antibodies. As can be observed in Figure 4B, adherence of *L. salivarius* Lv72 gradually increased such that 24 h after incubation the HeLa cells were completely covered, as would be expected from the enhancement of *oppA* expression that occurred upon mixing the two cell types.

## Discussion

*Lactobacilli* are important members of the autochthonous microbiota, colonizing a variety of internal human cavities. In addition, *Lactobacilli* constitute the bulk of the human vaginal microbiota, this clearly being a very recent evolutionary accomplishment given that it does not colonize the vagina of any other mammal, not even the large primates (32). A variety of bacterial adhesins and eukaryotic receptors have been found to mediate the attachment of *Lactobacilli* to the mucosal surfaces. Among them, the mutual recognition between OppA and GAGs that are part of the epithelial glycocalix appears to play a significant role and several lines of evidence support this (23, 25, 26). The benefits linked to the mutualism derived from the interaction between *Lactobacilli* and the human mucosa suggest that both participants might have evolved mechanisms to strengthen their initial casual contact in order to stabilize their symbiotic relationship.

The existence of an inducible system to promote adherence was evident for the *Lactobacilli*, as observed in the enormous increase in *oppA* transcription upon contact of the bacterium with HeLa cell layers. In addition, this induction appeared to be long-lived in that it remained at the same level for 6 h post-contact, and even 24 h after co-incubation the generation of *oppA*-specific RNA was enhanced several-fold with respect to that of the pure *L. salivarius* Lv72 cultures used as controls. This finding suggests that induction of *oppA* might last for as long as the bacterium and the mucosal cells remain together. On the other hand, the initial promotion of the attachment appears to be delayed, despite the fast and intense transcriptional response of the bacterium, because it was seen to develop gradually over a period of several hours. This indicates that translation of the transcripts and export of the resulting polypeptides to the bacterial surface is a slow process. In this respect, it should be highlighted that *oppA* was initially described as the substrate recognition component of an oligopeptide ABC-transporter comprised of two additional and homologous integral membrane proteins (OppB and OppC), which form the translocation pore, and two cytoplasm proteins (OppD and OppF), which drive the transport process through binding and hydrolysis of ATP (24). It could be that export of OppA is dependent on the formation of the ABC-transport complex, which would probably account for the delay in its accumulation on the bacterial surface. This might have some advantages for *Lactobacilli*, since they are multiauxotrophic and could benefit from the putative increment of oligopeptide internalization, especially in a protein rich environment such as the epithelial glycocalyx. Alternatively, OppA might be secreted, which raises the question of how it would remain bound to the bacterial wall and exposed to the environment. Moreover, the Opp-ABC transporters have been implicated in the recognition of the oligopeptides involved in quorum sensing (bacterial pheromones) (33) that mediate diverse *Lactobacilli*-driven processes, some of which, such as the production of bacteriocins (34), the ability to form biofilms (35), and adherence to epithelial surfaces (36) might contribute to their beneficial role.

Most HSPGs appear associated with the cell surface, the two most important gene families being syndecans and glypicans, although other minor or “part time” species, such as betaglycan and CD44v3 isoform, may also appear. Apart from serglycin, which is located intracellularly, the other species are closely associated with the surface of many cell types, being located principally in the pericellular region or in basement membranes (37). *Lactobacilli* adhesion to HeLa cells induces a decrease in transcription in more than 50% of the HSPG species expressed. This reduction particularly affects the syndecans, which constitute the main group of molecules present on the cell surface of HeLa cells the isoforms of 3 of which appear underexpressed. This result is particularly interesting because in certain studies it has been described that syndecans, acting cooperatively, are primarily responsible for bacterial adhesion, as occurs in gastric epithelial cells and macrophages (38) and in corneal epithelial cells (39). Another implication of this result is that, given that HS polysaccharides generally occur as HSPG, the decrease observed in core proteins should cause a decrease in the levels these saccharide chains on the cell surface and in the pericellular region.

Transcripts for 36 out of 47 genes involved in the biosynthesis of GAG chains could be detected, and 17 of them (47%) showed significant repression when the *Lactobacilli* were placed in contact with HeLa cell cultures. The genes affected are implicated in all production steps, i.e., synthesis of tetrasaccharide linker, initiation and polymerization of GAG chains, and fine-tuning the structure of the final macromolecule.

Although the organization and regulation of the synthesis of GAG chains is largely unknown, it is known that the expression levels of the enzymes involved play an essential role. It has also been proposed that these enzymes be grouped together in a hypothetical complex structure, referred to as a gagosome, which it is also hypothesized may contain regulatory proteins of an unknown nature (40). In addition, it is also known that regulation exists that is produced by some of the biosynthetic enzymes themselves, by the availability of precursors, or by enzymatic mechanisms such as phosphorylation of the xylose residue present in the binding tetrasaccharide (41). Our results show a particularly interesting reduction in the transcription of certain enzymes that are essential in the initiation and polymerization of GAG chains, such as those responsible for the initiation of HS chains (EXTL2 and EXTL3), the CS polymerization factor (CHPF) and, notably, those responsible for the synthesis of the tetrasaccharide linker and its phosphorylation (FAM20B). These data, together with the decrease in the transcription of the core proteins, strongly suggest the existence of a reduction in the synthesis of GAG chains is induced by the union of the microorganism. However, the GAGs had higher molecular masses, which might help the initial interaction of the glycocalyx components with the colonizing *Lactobacilli*. Nevertheless, the generalized gene-repression leading to the observed decrease in superficial GAGs seems puzzling, especially considering the extraordinary expression increase of *oppA* following the interaction of the two cell classes and the well-known mutualistic effect exerted by *Lactobacilli* colonization of the mucosae. However, this apparent paradox can be understood when the ecological conditions under which these two cell types live are taken into consideration. *Lactobacilli* colonize the external environment, where overexpression of *oppA* might not be as useful as in the internal cavities, where OppA is the anchor that enables fixation to the mucosae. On the other hand, from birth, the epithelial cells that form the walls of those cavities are covered by evolving microbiotas (42, 43). Consequently, when these cells are grown in pure culture, they are confronted by an unexpected and potentially stressful situation. This may induce overexpression of the genes involved in PG biosynthesis in order to maximize the possibility of attachment by beneficial microbes that might be present in lumen fluid. Once the interaction is established, the epithelial cells may then relax their expression of the PG biosynthesis determinants to a level which simply maintains contact between its own glycocalyx and that of the microbe and, thus, the advantages conferred by their mutual association.

In conclusion, the results of the present work show that the adhesion of *Lactobacillus salivarius* Lv72 to HeLa cell cultures induces alterations in the expression levels of certain molecules involved in the process. These alterations involve overexpression of the *Lactobacilli* adhesin OppA, and also of genes encoding some PG core proteins, as well as genes encoding some of the enzymes involved in the synthesis of the GAG chains. The main modifications affect glycosyltransferases, which are responsible for the synthesis of GAGs, but other genes are also affected. These mechanisms are probably part of the communication system between epithelial cells and the microbiota.

## Data Availability Statement

All datasets generated for this study are included in the article/Supplementary Material.

## Author Contributions

CM and IF-V carried out most of the experiments. JS and LQ co-ordinated the study and drafted the manuscript. All authors have read and approved the final manuscript.

### Conflict of Interest

The authors declare that the research was conducted in the absence of any commercial or financial relationships that could be construed as a potential conflict of interest.

## Abbreviations

CS: chondroitin sulfate
GAG: glycosaminoglycan
HS: heparan sulfate
HSPG: Heparan sulfate proteoglycan.
