# Supplementary material for: Adherence of Lactobacillus salivarius to HeLa Cells Promotes Changes in the Expression of the Genes Involved in Biosynthesis of Their Ligands
Source: Front Immunol. 2020 Jan 9;10:3019. doi: 10.3389/fimmu.2019.03019 (PMC6962182; doi:10.3389/fimmu.2019.03019)
Supplement: Supplementary file 1 [file Data_Sheet_1.PDF]

Supplemental table 1

qRT-PCR primer sequences

Gene	Gene ID	Primer sequence
SDC1	6382	F 5'- CTCAGGTGCAGGTGCTTTG<br>R 5'- CTGCGTGTCCTTCCAAGTG
SDC2	6383	F 5'- GATGACGATGACTACGCTTCTG<br>R 5'- TGGAAGTGGTCGAGATGTTG
SDC3	9672	F 5'- CTCCTTTCCCGATGATGAAC<br>R 5'- CGACTCCTGCTCGAAGTAGC
SDC4	6385	F 5'- GGCAGGAATCTGATGACTTTG<br>R 5'- TCTAGAGGCACCAAGGGATG
GPC1	2817	F 5'- CATCGGGTGTGGAGAGTG<br>R 5'- TGAGCGTGTCCCTGTTGTC
GPC2	221914	F 5'- CTGGGACACGACCTGGAC<br>R 5'- GCCATCCAGTCATCTGCATAC
GPC3	2719	F 5'- CTGCTTCAGTCTGCAAGTATGG<br>R 5'- GTGGAGTCAGGCTTGGGTAG
GPC4	2239	F 5'- AGTGTGGTCAGCGAACAGTG<br>R 5'- CAAACATATCATTCAGGGATTTCTC
GPC5	2262	F 5'- GCCGCCCTGTAAGAACAC<br>R 5'- TCATTCCATGCTTCTCTTTGC
GPC6	10082	F 5'- CCAGGCATAAGAAATTTGACG<br>R 5'- CATGTACAGCATGCCATAGGTC
PRCAN	3339	F 5'- TGGACACATTCGTACCTTTCTG<br>R 5'- CACTGCCCAGGTCGTCTC
AGRN	375790	F 5'- ACTGTGTCTGCCCGATGC<br>R 5'- GACACTCGTTGCCGTATGTG
COL18A1	80781	F 5'- GTACAAGGGAGAGATTGGCTTTC<br>R 5'- TTTCTCTCCTTTCAATCCGTTC
TGFBR3	7049	F 5'- AGTGTGAGCTGACGCTGTGTA<br>R 5'- GGGCTTAGTGAACGTCTTCTTATTC
CD44 (v3)	960	F 5'- TGGGAGCCAAATGAAGAAAATGAA<br>R 5'- TGGTTGAAATGGTGCTGGAGA
SRGN	5552	F 5'- TCCTGGTTCTGGAATCCTCA<br>R 5'- TCTTGTTGGATTCACCTGGAA
XYLT1	64131	F 5'- ACTACCCCATCAGGACAAATGA<br>R 5'- CTGCTTCCGAATGAACCTTG
XYLT2	64132	F 5'- AGGGCCTGGTAGTGTGGAG<br>R 5'- TGAACTGTCTGTGTCCTTGGAA
FAM20B	9917	F 5'- TCTGCAGAAGCACCGTCA<br>R 5'- CAGCTGTGTCAATGATGTCCA
B4GALT7	11285	F 5'- GCGAGGACGACGAGTTCTAC<br>R 5'- CAGGTGGCGAAATGTCTTGTA
B3GALT6	126792	F 5'- CACGTGGCCTTCGAGTTC<br>R 5'- CCGAGAAGAAGCCCCAGTA
B3GAT1	27087	F 5'- TGGTGAATGAGGGCAAGAA<br>R 5'- CTTAGGAGTCGGCCTTGGA
B3GAT2	135152	F 5'- GCTGACGACGACAACACCTA<br>R 5'- CGGTGTACCAGCCAACAAC
B3GAT3	26229	F 5'- GAAGAACGTGTTTCTCGCCTAC<br>R 5'- CCTCAGATCCTTCTGCCGTA
EXTL1	2134	F 5'- GATGAGAGGCTCCCACTTCA<br>R 5'- CCTCCAGAGTGGTATGGATGA
EXTL2	2135	F 5'- TGAACTGGAAACCAATGCAG<br>R 5'- AGGAAATTGCTGCCAAACTG
EXTL3	2137	F 5'- CTCCGCCATGACGAAATC<br>R 5'- AGTTGGAGTTGTAGAGCCAGGA
EXT1	2131	F 5'- GAGACAATGATGGGACAGACTTC

		R 5’- CTCTGTCGCTGGGCAAAG
EXT2	2132	F 5’- CTGGGACCATGAGATGAATA<br>R 5’- GATATCCCCAGGCATTTTGTA
CSGALNACT1	55790	F 5’- GGAGACCCTGAACAATCCTG<br>R 5’- GCCGTTTGAATTCGTGTTTG
CSGALNACT2	55454	F 5’- GCCATTGTTTATGCCAACCA<br>R 5’- ATCCACCAATGGTCAGGAAA
CHSY1	22856	F 5’- GCCCAGAAATACCTGCAGAC<br>R 5’- GCACTACTGGAATTGGTACAGATG
CHPF	79586	F 5’- GGTGCACTATAGCCATCTGGA<br>R 5’- GGCACTTCGGAAATGAGG
CHSY3	337876	F 5’- GACTCAGTGTGTCTGGTCTTACG<br>R 5’- TTGCTATTGTGAAGGTCTTGGA
NDST1	3340	F 5’- CTGCCCTCTACCTGTTCCTG<br>R 5’- AACTGGATCTCCTCAAAGGTCTC
NDST2	8509	F 5’- CAAGAGCTGCGTACCAACC<br>R 5’- GAGGGTCCGTGTGTAGTTCAG
NDST3	9348	F 5’- CCTTGCAGAAGAGATGTTTGG<br>R 5’- GTAGCAGGATCAGTTCTTAGTTGTTG
NDST4	64579	F 5’- GACATTGGGCTCCATCTGAC<br>R 5’- GCTGCTGTCCATCAATAATTAGC
GLCE	26035	F 5’- TGTGGAAGTCCGAGACAGAG<br>R 5’- CTGGATTGGATAGAAATAGCCTTG
HS2ST1	9653	F 5’- TGGAGATGATTATAGACCAGGGTTAC<br>R 5’- GCTATGGCCACAGAAGAACG
HS6ST1	9394	F 5’- GCAGGGAGTGGAGCTAACAG<br>R 5’- AACAGTTCCAGTTCCCGAAA
HS6ST2	90161	F 5’- CGGTGCGATCTTCTCCAA<br>R 5’- AGGACGATCACGGCAAATAG
HS6ST3	266722	F 5’- CAACCACAGCCACACCAG<br>R 5’- CTTCTTCCATCACACATATGAAGAG
HS3ST1	9957	F 5’- CAGCCAGATGCCCTTCTC<br>R 5’- AGACTCGCTCAGGCACTTTG
HS3ST2	9956	F 5’- GATTGGTACAGGAGCCTGATG<br>R 5’- GGAGCCTCTTGAGTGACAAAG
HS3ST3A1	9955	F 5’- GGCCGAGAGAACCTGAACTC<br>R 5’- CGAGCGACAGTGACTTCCA
HS3ST3B1	9953	F 5’- GCAGATCTTGCCTCGATGTC<br>R 5’- GCGCACGAGTACAGGAACATA
HS3ST4	9951	F 5’- TAGAGCCGCACTTCTTCGAC<br>R 5’- GGTTATTTGCCCATCCAAAG
HS3ST5	222537	F 5’- CATCCGGCAGTAGTCAAAGC<br>R 5’- TTGTGATTTGCTGAGGGTAGG
HS3ST6	64711	F 5’- GCCCTGCTGGAGTTTCTG<br>R 5’- GCGCTCGTAGCACCTGTC
SULF1	23213	F 5’- CCAGCAGAAGCCAAAGAAAG<br>R 5’- GAACGTGTCTGCCGAGTATG
SULF2	55959	F 5’- GCCTGCAAGAGAAGGACAAG<br>R 5’- AGCAGCTTGCGGAGTTTC
CHST11	50515	F 5’- CGCTGCTGGAAGTGATGA<br>R 5’- AGGATAAAGGATCCCAAGCAA
CHST12	55501	F 5’- GTAGCCGACAAATCCTTCCA<br>R 5’- ACCGGTTTACCTCTGACTTGAC
CHST13	166012	F 5’- CCGGCATTTGGAAACAGA<br>R 5’- TCCAGGTCATAGAGCTTCTGC
CHST14	113189	F 5’- CCACTGCCTAATGTCACCAA<br>R 5’- ATGACAGGCAGAAGCACAGA
CHST15	51363	F 5’- GTGCCAGGAATAAAGTTCAACA<br>R 5’- CACTGGATAAGTCCCGAGTGA
CHST3	9469	F 5’- TGCACAGCCTGAAGATGAGA<br>R 5’- CAGCTTGTCTGAGACCCTTGA

CHST7	56548	F 5'- GATCCGGGTCAGTCACCA<br>R 5'- GACAGATTGCCCCCACAG
DSE	29940	F 5'- GTCCAGAGGCACTTCAACATC<br>R 5'- AGTCCGCAATAGCCACAGTC
UST	10090	F 5'- ACCATGGACCACCTCCTAGTAA<br>R 5'- CACACTTGCCTACCCTGTTGTA
HPSE	10855	F 5'- CCTTGCTATCCGACACCTTTG<br>R 5'- TATTCTTTGGAGCAGGAAACTACC
HPSE2	60495	F 5'- CACCCTGATGTTATGCTGGAG<br>R 5'- TCCAGAGCAATCAGCAAAGTTA
GAPDH (human)	2597	F 5'- GAGTCCACTGGCGTCTTCAC<br>R 5'- GTTCACACCCATGACGAACA
gapA (GAPDH *Lactobacillus salivarius*)	3978001	F 5'- GCCGTCACCCTTACCTACAA<br>R 5'- CAACCCATCATTCGGTTACA
